# Supplementary material for: Types and anatomical locations of injuries among mountain bikers and hikers: A systematic review
Source: PLoS One. 2023 Aug 30;18(8):e0285614. doi: 10.1371/journal.pone.0285614 (PMC10468092; doi:10.1371/journal.pone.0285614)
Supplement: S1 Checklist — (DOCX) [file pone.0285614.s001.docx]

| **Section and Topic** | **Item #** | **Checklist item** | **Location where item is reported** |
| --- | --- | --- | --- |
| **TITLE** | | |  |
| Title | 1 | Identify the report as a systematic review. | Yes – title |
| **ABSTRACT** | | |  |
| Abstract | 2 | See the PRISMA 2020 for Abstracts checklist. | Yes – completed and compliant (available on request) |
| **INTRODUCTION** | | |  |
| Rationale | 3 | Describe the rationale for the review in the context of existing knowledge. | Yes – throughout introduction |
| Objectives | 4 | Provide an explicit statement of the objective(s) or question(s) the review addresses. | Yes – AIM on page 5 |
| **METHODS** | | |  |
| Eligibility criteria | 5 | Specify the inclusion and exclusion criteria for the review and how studies were grouped for the syntheses. | Yes – methods page 5/6 |
| Information sources | 6 | Specify all databases, registers, websites, organisations, reference lists and other sources searched or consulted to identify studies. Specify the date when each source was last searched or consulted. | Yes - methods (data sources and search strategy) page 6 |
| Search strategy | 7 | Present the full search strategies for all databases, registers and websites, including any filters and limits used. | Yes – available in separate document for each database (included in submission) |
| Selection process | 8 | Specify the methods used to decide whether a study met the inclusion criteria of the review, including how many reviewers screened each record and each report retrieved, whether they worked independently, and if applicable, details of automation tools used in the process. | Yes - detailed in methods page 6/7. 3 reviewers, working independently and tool was rayyan |
| Data collection process | 9 | Specify the methods used to collect data from reports, including how many reviewers collected data from each report, whether they worked independently, any processes for obtaining or confirming data from study investigators, and if applicable, details of automation tools used in the process. | N/A – data was not collected for synthesis due to reasons explained in the report. Where data was taken for descriptive review described in methods page 6/7 |
| Data items | 10a | List and define all outcomes for which data were sought. Specify whether all results that were compatible with each outcome domain in each study were sought (e.g. for all measures, time points, analyses), and if not, the methods used to decide which results to collect. | Yes – injury location and injury type detailed in methods on page 4-6. Detailed explanation given in discussion regarding heterogeneity of results and inability to compare intra activity and inter activity |
|  | 10b | List and define all other variables for which data were sought (e.g. participant and intervention characteristics, funding sources). Describe any assumptions made about any missing or unclear information. | Yes – incidence rates also sought and discussed but not able to synthesise due to above reasons. Described in results but not synthesised |
| Study risk of bias assessment | 11 | Specify the methods used to assess risk of bias in the included studies, including details of the tool(s) used, how many reviewers assessed each study and whether they worked independently, and if applicable, details of automation tools used in the process. | Yes – discussed in methods page 7. JBI checklist for case series was utilised. 2 reviewers PJB and HT worked independently. No automation tools used |
| Effect measures | 12 | Specify for each outcome the effect measure(s) (e.g. risk ratio, mean difference) used in the synthesis or presentation of results. | Partial – presented as n and % in aggregated results tables on page 12,13,17,18 |
| Synthesis methods | 13a | Describe the processes used to decide which studies were eligible for each synthesis (e.g. tabulating the study intervention characteristics and comparing against the planned groups for each synthesis (item #5)). | N/A due to no synthesis possible |
|  | 13b | Describe any methods required to prepare the data for presentation or synthesis, such as handling of missing summary statistics, or data conversions. | Yes – any anomalous data is explained in the results tables mentioned above utilising superscript ^a^ or * |
|  | 13c | Describe any methods used to tabulate or visually display results of individual studies and syntheses. | YES – results aggregated from each paper into simplified bodily location. I.e. if a paper presented injuries to abdomen and chest these were aggregated to torso. If it gave ankle/knee/femur these were aggregated to lower limb. This is then presented as n and % in aggregated results tables on page 8,9,11,12. This method is detailed in last paragraph ‘injury location’ on page 7 |
|  | 13d | Describe any methods used to synthesize results and provide a rationale for the choice(s). If meta-analysis was performed, describe the model(s), method(s) to identify the presence and extent of statistical heterogeneity, and software package(s) used. | N/A |
|  | 13e | Describe any methods used to explore possible causes of heterogeneity among study results (e.g. subgroup analysis, meta-regression). | Yes discussed throughout regarding the heterogenous definition of injury an the way injuries were recorded. |
|  | 13f | Describe any sensitivity analyses conducted to assess robustness of the synthesized results. | N/A |
| Reporting bias assessment | 14 | Describe any methods used to assess risk of bias due to missing results in a synthesis (arising from reporting biases). | N/A |
| Certainty assessment | 15 | Describe any methods used to assess certainty (or confidence) in the body of evidence for an outcome. | N/A |
| **RESULTS** | | |  |
| Study selection | 16a | Describe the results of the search and selection process, from the number of records identified in the search to the number of studies included in the review, ideally using a flow diagram. | Yes – page 8 in flow diagram (not included in paper due to sizing but sent separately as document to be included) |
|  | 16b | Cite studies that might appear to meet the inclusion criteria, but which were excluded, and explain why they were excluded. | Partial – too many to discuss individually so two were referenced and explained in the discussion section using the statement “*many studies reporting injuries to trail users could not be included due to the use of questionnaire-based injury identification and the inherent recall and selection bias that comes with this type of recruitment method”* |
| Study characteristics | 17 | Cite each included study and present its characteristics. | Yes – throughout and in tables |
| Risk of bias in studies | 18 | Present assessments of risk of bias for each included study. | N/A – discussed broadly in the quality assessment section on page 5 but due to no synthesis and it being descriptive we have not summarised the results of the RoB assessments |
| Results of individual studies | 19 | For all outcomes, present, for each study: (a) summary statistics for each group (where appropriate) and (b) an effect estimate and its precision (e.g. confidence/credible interval), ideally using structured tables or plots. | Yes – 4 results tables 2 for MTB and 2 for hiking showing this |
| Results of syntheses | 20a | For each synthesis, briefly summarise the characteristics and risk of bias among contributing studies. | N/A |
|  | 20b | Present results of all statistical syntheses conducted. If meta-analysis was done, present for each the summary estimate and its precision (e.g. confidence/credible interval) and measures of statistical heterogeneity. If comparing groups, describe the direction of the effect. | N/A |
|  | 20c | Present results of all investigations of possible causes of heterogeneity among study results. | Yes – discussed throughout regarding the broad use of the term injury and how the data was collected |
|  | 20d | Present results of all sensitivity analyses conducted to assess the robustness of the synthesized results. | N/A |
| Reporting biases | 21 | Present assessments of risk of bias due to missing results (arising from reporting biases) for each synthesis assessed. | N/A |
| Certainty of evidence | 22 | Present assessments of certainty (or confidence) in the body of evidence for each outcome assessed. | N/A |
| **DISCUSSION** | | |  |
| Discussion | 23a | Provide a general interpretation of the results in the context of other evidence. | Yes – throughout discussion and broken into mountain biking and hiking |
|  | 23b | Discuss any limitations of the evidence included in the review. | Yes – multiple limitations discussed regarding data collection, definitions and the retrospective nature of the data |
|  | 23c | Discuss any limitations of the review processes used. | Yes – lack of ability to synthesis data discussed throughout and specific section towards end of discussion regarding the limitations of the work |
|  | 23d | Discuss implications of the results for practice, policy, and future research. | Yes – implications for practice made regarding need for concussion protocol in competitive mountain biking, need for a standardised definition of injury in sports epidemiology |
| **OTHER INFORMATION** | | |  |
| Registration and protocol | 24a | Provide registration information for the review, including register name and registration number, or state that the review was not registered. | Yes - page 3 PROSPERO CRD42021229623 |
|  | 24b | Indicate where the review protocol can be accessed, or state that a protocol was not prepared. | Yes – registered with Prospero available online |
|  | 24c | Describe and explain any amendments to information provided at registration or in the protocol. | Yes – Some amendments needed with reviewer due to staff availability and use of the JBI checklist and not downs and black due to all papers being case series and JBI checklist having better suitability. |
| Support | 25 | Describe sources of financial or non-financial support for the review, and the role of the funders or sponsors in the review. | N/A |
| Competing interests | 26 | Declare any competing interests of review authors. | N/A |
| Availability of data, code and other materials | 27 | Report which of the following are publicly available and where they can be found: template data collection forms; data extracted from included studies; data used for all analyses; analytic code; any other materials used in the review. | N/A due to no synthesis and everything else has been made available in the report |

*From:*  Page MJ, McKenzie JE, Bossuyt PM, Boutron I, Hoffmann TC, Mulrow CD, et al. The PRISMA 2020 statement: an updated guideline for reporting systematic reviews. BMJ 2021;372:n71. doi: 10.1136/bmj.n71

For more information, visit: <http://www.prisma-statement.org/>
