# Supplementary material for: Types and anatomical locations of injuries among mountain bikers and hikers: A systematic review
Source: PLoS One. 2023 Aug 30;18(8):e0285614. doi: 10.1371/journal.pone.0285614 (PMC10468092; doi:10.1371/journal.pone.0285614)
Supplement: S1 Table — (DOCX) [file pone.0285614.s002.docx]

| Line | Scopus (executed 01.03.2022) | No. |
| --- | --- | --- |
| 1 | TITLE-ABS-KEY ( "trail run*" OR "off-road run*" OR "fell run*" OR "mountain run*" OR hiking OR hike* OR trek* OR "mountain bik*" OR "mtb" OR "mountain bicycl*" OR "mountain cycl*" OR "off-road cycl*" ) AND TITLE-ABS-KEY ( injur* OR trauma OR accident* OR morbidity OR mortality ) |  |

| Line | Cochrane (executed 01.03.2022) | No. |
| --- | --- | --- |
| 1 | ("trail run*" OR "off-road run*" OR "fell run*" OR "mountain run*" OR hiking OR hike* OR trek* OR "mountain bik*" OR "mtb" OR "mountain bicycl*" OR "mountain cycl*" OR "off-road cycl*" ) AND ( injur* OR trauma OR accident* OR morbidity OR mortality ) |  |

| Line | Pubmed (executed 01.03.2022) | No. |
| --- | --- | --- |
| 1 | ("trail run*" OR "off-road run*" OR "fell run*" OR "mountain run*" OR hiking OR hike* OR trek* OR "mountain bik*" OR "mtb" OR "mountain bicycl*" OR "mountain cycl*" OR "off-road cycl*" ) AND ( injur* OR trauma OR accident* OR morbidity OR mortality ) |  |

| Line | ProQuest (executed 01.03.2022) | No. |
| --- | --- | --- |
| 1 | noft(("trail run" OR "trail runner" OR "trail runners" OR "trail running" OR "trail runs") OR "off-road run*" OR ("fell runner" OR "fell runners" OR "fell running") OR ("mountain run" OR "mountain runner" OR "mountain running" OR "mountain runs") OR hiking OR hike* OR trek* OR ("mountain bike" OR ("mountain bike" OR "mountain bikemen" OR "mountain biker" OR "mountain bikers" OR "mountain bikes" OR "mountain biking") OR "mountain biker" OR "mountain bikers" OR "mountain bikes" OR "mountain biking") OR "mtb" OR "mountain bicycl*" OR "mountain cycl*" OR "off-road cycl*" ) AND ( injur* OR trauma OR accident* OR morbidity OR mortality ) AND PEER(yes) |  |

| Line | CINAHL (executed 01.03.2022) | No. |
| --- | --- | --- |
| 1 | "trail run*" OR "off-road run*" OR "fell run*" OR "mountain run*" OR hiking OR hike* OR trek* OR "mountain bik*" OR "mtb" OR "mountain bicycl*" OR "mountain cycl*" OR "off-road cycl*" |  |
| 2 | injur* OR trauma OR accident* OR morbidity OR mortality |  |
| 3 | 1 and 2 |  |
