# Supplementary material for: Types and anatomical locations of injuries among mountain bikers and hikers: A systematic review
Source: PLoS One. 2023 Aug 30;18(8):e0285614. doi: 10.1371/journal.pone.0285614 (PMC10468092; doi:10.1371/journal.pone.0285614)
Supplement: S1 Appendix — (DOCX) [file pone.0285614.s003.docx]

|  | Question | 1 | 2 | 3 | 4 | 5 | 6 | 7 | 8 | 9 | 10 |
| --- | --- | --- | --- | --- | --- | --- | --- | --- | --- | --- | --- |
| First Author |  |  |  |  |  |  |  |  |  |  |  |
| CYCLING STUDIES |  |  |  |  |  |  |  |  |  |  |  |
| Ashwell[1] |  | y | y | y | y | y | y | y | y | y | n/a |
| Cajani[2] |  | y | u | u | y | y | n | u | y | y | u |
| Carmont[3] |  | u | n | n | u | u | n | n | y | n | n/a |
| Chow[4] |  | y | y | y | y | y | y | y | y | y | y |
| Jeys[5] |  | y | y | y | y | y | y | n | y | y | y |
| Kim[6] |  | y | y | y | y | y | y | u | y | y | y |
| Kotlyar[7] |  | y | y | y | y | y | n | y | y | y | y |
| Kronisch[8] |  | y | y | y | y | y | y | y | y | y | y |
| Kronisch[9] |  | y | y | y | y | y | n | y | y | y | n |
| Kronisch[10] |  | n | y | y | y | y | n | n | y | y | y |
| Nelson[11] |  | y | y | n | y | y | y | y | y | n | y |
| Palmer[12] |  | y | y | y | y | y | n | y | y | y | y |
| Rivara[13] |  | y | n | n | y | n | y | y | y | y | y |
| Roberts[14] |  | y | y | y | y | y | y | y | y | y | y |
| Romanow[15] |  | y | y | y | y | y | y | y | y | y | y |
| Saragaglia[16] |  | y | y | y | y | y | y | n | y | n/a | n |
| Taylor[17] |  | y | y | y | y | y | n | y | y | y | n |
| HIKING STUDIES |  |  |  |  |  |  |  |  |  |  |  |
| Faulhaber[18] |  | y | y | y | y | y | y | y | y | n | y |
| Chu[19] |  | y | y | y | y | y | y | n | y | n | n |
| scheer[20] |  | u | y | y | u | u | y | n | y | u | n/a |
| Vernillo[21] |  | y | y | y | n | n | y | y | y | y | y |
| wong[22] |  | y | y | y | n | n | y | y | y | y | n/a |
| McClean[23] |  | n | u | u | u | u | n | n | y | u | n/a |
| Gasser[24] |  | y | u | y | y | y | n | n | y | y | n |

|  | Question | 1 | 2 | 3 | 4 | 5 | 6 | 7 | 8 | 9 | 10 |
| --- | --- | --- | --- | --- | --- | --- | --- | --- | --- | --- | --- |
| First Author |  |  |  |  |  |  |  |  |  |  |  |
| CYCLING STUDIES |  |  |  |  |  |  |  |  |  |  |  |
| Ashwell[1] |  | y | y | y | y | y | y | y | y | y | y |
| Cajani[2] |  | y | y | y | y | y | n | y | y | y | u |
| Carmont[3] |  | y | n | u | y | u | n | n | y | n | u |
| Chow[4] |  | y | y | y | y | y | n | y | y | y | y |
| Jeys[5] |  | y | y | y | y | y | n | n | y | y | y |
| Kim[6] |  | y | y | y | y | y | y | u | y | y | n |
| Kotlyar[7] |  | y | y | y | y | y | y | y | y | y | y |
| Kronisch[8] |  | y | y | y | y | y | y | y | y | y | y |
| Kronisch[9] |  | y | y | y | y | y | n | y | y | n | y |
| Kronisch[10] |  | n | y | y | y | y | n | y | y | y | y |
| Nelson[11] |  | y | y | y | y | y | y | y | y | y | y |
| Palmer[12] |  | y | y | y | y | y | y | y | y | y | y |
| Rivara[13] |  | y | y | y | y | n | y | y | y | y | y |
| Roberts[14] |  | y | y | y | y | y | y | y | y | y | y |
| Romanow[15] |  | y | y | y | y | y | y | y | u | y | y |
| Saragaglia[16] |  | y | y | y | y | y | y | y | y | y | y |
| Taylor[17] |  | y | y | y | y | n | n | y | y | y | y |
| HIKING STUDIES |  |  |  |  |  |  |  |  |  |  |  |
| Faulhaber[18] |  | y | y | y | y | y | y | y | y | y | y |
| Chu[19] |  | y | y | y | y | y | y | n | y | n | u |
| scheer[20] |  | n | y | y | u | u | n | u | n/a | n | n/a |
| Vernillo[21] |  | y | y | y | n | n | y | n | n/a | n | y |
| wong[22] |  | y | n | y | n | n | n | n | y | n/a | n/a |
| McClean[23] |  | n | u | u | y | y | n | n | y | u | y |
| Gasser[24] |  | y | y | u | y | y | n | n | y | y | y |

1. Ashwell Z, McKay MP, Brubacher JR, Gareau A. The epidemiology of mountain bike park injuries at the Whistler Bike Park, British Columbia (BC), Canada. Wilderness Environ Med. 2012;23(2):140-5.

2. Cajani S, Fischer H, Pietsch U. Emergency service care of mountain bike elite races: Rescue concept and analysis of 5 years of world cup elite cross-country/downhill and marathon stage races. Anaesthesist. 2022;71(1):59-64.

3. Carmont MR, Daynes R, Sedgwick DM. The impact of an extreme sports event on a district general hospital. Scott Med J. 2005;50(3):106-8.

4. Chow TK, Kronisch RL. Mechanisms of injury in competitive off-road bicycling. Wilderness & Environmental Medicine. 2002;13(1):27-30.

5. Jeys LM, Cribb G, Toms AD, Hay SM. Mountain biking injuries in rural England. Br J Sports Med. 2001;35(3):197-9.

6. Kim PT, Jangra D, Ritchie AH, Lower ME, Kasic S, Brown DR, et al. Mountain biking injuries requiring trauma center admission: a 10-year regional trauma system experience. Journal of Trauma-Injury Infection & Critical Care. 2006;60(2):312-8.

7. Kotlyar S. Cycling Injuries in Southwest Colorado: A Comparison of Road vs Trail Riding Injury Patterns. Wilderness & Environmental Medicine. 2016;27(2):316-20.

8. Kronisch RL, Pfeiffer RP, Chow TK, Hummel CB. Gender differences in acute mountain bike racing injuries. Clin J Sport Med. 2002;12(3):158-64.

9. Kronisch RL, Chow TK, Simon LM, Wong PF. Acute injuries in off-road bicycle racing. The American Journal of Sports Medicine. 1996;24(1):88.

10. Kronisch RL, Pfeiffer RP, Chow TK. Acute injuries in cross-country and downhill off-road bicycle racing. Medicine & Science in Sports & Exercise. 1996;28(11):1351-5.

11. Nelson NG, McKenzie LB. Mountain biking-related injuries treated in emergency departments in the United States, 1994-2007. Am J Sports Med. 2011;39(2):404-9.

12. Palmer D, Ball C, Florida-James G. Enduro mountain biking injuries during the enduro world series: a two-season prospective study. British Journal of Sports Medicine. 2020;54.

13. Rivara FP, Thompson DC, Thompson RS, Rebolledo V. Injuries involving off-road cycling. J Fam Pract. 1997;44(5):481-5.

14. Roberts DJ, Ouellet JF, Sutherland FR, Kirkpatrick AW, Lall RN, Ball CG. Severe street and mountain bicycling injuries in adults: a comparison of the incidence, risk factors and injury patterns over 14 years. Can J Surg. 2013;56(3):E32-8.

15. Romanow NT, Hagel BE, Nguyen M, Embree T, Rowe BH. Mountain bike terrain park-related injuries: an emerging cause of morbidity. International Journal of Injury Control & Safety Promotion. 2014;21(1):29-46.

16. Saragaglia D, Favarel G, Banihachemi JJ. Downhill mountain biking traumatology: Prospective epidemiological study of 138 cases totaling 178 lesions. Journal de Traumatologie du Sport. 2020;37(3):139-44.

17. Taylor NB, Ranse J. Epidemiology of injuries at the Australian 24 hour mountain bike championships. Australasian Journal of Paramedicine. 2013;10(1).

18. Faulhaber M, Pocecco E, Niedermeier M, Ruedl G, Walter D, Sterr R, et al. Fall-related accidents among hikers in the Austrian Alps: a 9-year retrospective study. BMJ Open Sport Exerc Med. 2017;3(1):e000304.

19. Chu WYC, Chong YC, Mok WY. Hiking-related orthopaedic injuries: Another epidemic during the COVID-19 pandemic. Journal of Orthopaedics, Trauma & Rehabilitation. 2021;28:1-4.

20. Scheer BV, Murray A. Al Andalus Ultra Trail: an observation of medical interventions during a 219-km, 5-day ultramarathon stage race. Clin J Sport Med. 2011;21(5):444-6.

21. Vernillo G, Savoldelli A, La Torre A, Skafidas S, Bortolan L, Schena F. Injury and Illness Rates During Ultratrail Running. Int J Sports Med. 2016;37(7):565-9.

22. Wong TW, Lau PF, Lau CC. Aeromedical evacuation of injured hikers in Hong Kong. World J Emerg Med. 2010;1(3):176-9.

23. McLean I. First Aid for Orienteering in Scotland. Scientific Journal of Orienteering. 1990;6(2):55-63.

24. Gasser B. Half of emergency calls in hikers are injuries from falls in 50-70 year-olds. Deutsche Zeitschrift fur Sportmedizin. 2019;70(9):209-14.
